# Editorial: Sustainable career development in the turbulent, boundaryless and internet age

**DOI:** 10.3389/fpsyg.2024.1358059

**Published:** 2024-07-01

**Authors:** Yongrok Choi, Shih-Chih Chen, Yin Ma, Athapol Ruangkanjanases

**Affiliations:** ^1^Department of International Trade, Inha University, Incheon, Republic of Korea; ^2^Department of Information Management, National Kaohsiung University of Science and Technology, Kaohsiung, Taiwan; ^3^School of Philosophy and Sociology, Lanzhou University, Lanzhou, China; ^4^Chulalongkorn Business School, Chulalongkorn University, Bangkok, Thailand

**Keywords:** career sustainability, 4th Industrial Revolution, globalization, governance, publicprivate partnership

## Introduction

The global economy has been experiencing turmoil because of the turbulent transition from the free trade regime of the World Trade Organization (WTO) to protectionism under diverse free trade zones. As the WTO created one global market under borderless competition, several developed countries have experienced severe economic recession, and thus, there have been ever-increasing trade conflicts among countries, represented by that between the US and China. Thus, instead of the free trade illusion, several countries have entered into bilateral or multilateral free trade agreements to survive the turmoil of global trade conflicts. During the transition, several countries have experienced economic depression, resulting in unemployment.

To overcome this major recession with unexpectedly increasing unemployment, several countries have promoted the venture type of new business, particularly for the young generation. Owing to various innovations, diverse job and business creations have arisen from this transition toward the 4th Industrial Revolution in the Internet age. One of the examples of this internet-supported job creation is short-form video platforms. Such platforms became a popular means of enhancing the value co-creation between brands and consumers and creating brand equity, and there are several ways to earn income from this value creation based on network management (Choi, 2017b). Chen et al. investigated the role and mechanism of value co-creation generated by consumer participation in short-form video platforms and found that the user's participation can boost brand equity and its resulting values. This value derived from Internet use has led to the era of Web 3.0. Users no longer just search for information or passively participate in content creation. They can even earn income from created content, and the brand equity is supported by several advertisement sponsors who are ready to participate in this value-creation network. Values from this Internet or human network are The source of sustainable development in the future. To improve the effectiveness of social media marketing and promotion of martial arts, Xie et al. examined the WeChat martial arts group users and found that social media marketing activities and user experience had a positive and significant effect on martial arts attitudes and subjective norms of value.

To determine the governance factors of this value-creation network in this transitional global economy, this Research Topic aims to reconstruct the value-creation network from the cracked boundaryless global market and identify the workable mechanism supported by the diverse internet revolution. Governance is defined as a workable mechanism for sustainable performance (Choi, 2017a). This Research Topic contains 25 papers on sustainable governance under the turbulent global economy on new issues on the emerging norm, enhanced approaches to the arguments, and unique insights into the implications and suggestions on the empirical findings. These papers are based on diverse perspectives of economy and business, IT engineering, environmental science, and multicultural geographical science. All contributing research touches on one of the issues of multidirectional governance but with unique arguments and challenges. Owing to the provocative status of this turbulent, transient regime, all the papers in Research Topic present very proactive and insightful arguments under the new norm of sustainable job creation in the Internet age.

## Workable new issues in the turbulent internet age

Under the WTO regime, despite the numerous benefits of borderless competition in the global market, consumers remain limited by significant trust factors, such as payment and lack of trust (Lin et al., 2022). To fill this missing link in the global economy, Research Topic raises several possible questions. To address the missing link in the mechanism of the “empty busy” behavior of grassroots cadres, Duan et al. highlighted four-dimensional paradoxes and that the “empty busy” behavior of grassroots cadres needs to be corrected from the four-dimensional logic of incentive, restraint, deep care, and strict control. Particularly, under the Paris Regime, all countries should make their best efforts to reduce greenhouse gas (GHG) emissions from economic activities. Nonetheless, this environmentally friendly commitment by government and industries may have several missing links, resulting in a lack of governance. For example, regarding the impact of corporate social responsibility (CSR) on green purchase intention, Tao et al. investigated empirical evidence from green building industries in Taiwan and found a missing link between green purchase intention (GPI) and green attitude (GA). To address this, they emphasized the mediating role of green trust (GT), implying that the workable mechanism should be based on the emotional network, even in the process of the mobile revolution. Thus, when we say that network management is important in the Internet age, this does not simply mean information sharing or human relationships in the virtual society. To develop a workable mechanism from this network, much stronger emotional ties are necessary, such as the Chinese concept of guanxì (Wang and Zhao, 2018). Here, Chinese guanxì can be defined as a stepwise interrelationship to share value from acquaintances via friendly trust to family loyalty (Choi, 2018).

Therefore, even in the mobile or AI revolution, reliable emotional sharing is required to create value from this network. This is the reason some users communicate every minute, whereas others communicate once a month or year. What makes this mechanism workable in the network? Several issues are addressed in this Research Topic. Wan and Cao examined the psychology of working theory to understand how decent work is related to employee wellbeing and established that the mediating effect of survival needs satisfaction was not significant; thus, social contribution should be based on satisfaction and self-determination needs (Wan and Cao). Even in an innovation ecosystem consisting of multiple agents and emerging as knowledge networks, one of the most important factors is not knowledge searching and sharing, but harmonizing this knowledge with other partners (Shi and Chen). In this Research Topic, they focused on the “Orchestrating process” of multi-agent knowledge ecosystems. Managers of makerspaces, as network managers for all participating agents, may collaboratively work on profit-sharing and are generally willing to help makers and startups incubate products and services. This implies that the creation of value in this workable network originates from innovation. The traditional market cannot satisfy all its partners because of its fixed profits; only a larger “pizza pie” can satisfy all the partners with a larger portion of their performance.

Therefore, the most important factor for the workable mechanism originates from this performance of innovation for all, and this win-win paradigm for network participants can enhance sustainable performance for all. Based on the educational virtual community context, Zhao and Shi evaluated the mediation mechanism of the sense of virtual community affecting user engagement and its boundary conditions. They found that effective commitment has a mediating role between the sense of virtual community and user engagement, whereas perceived support has a moderating role in the process of effective commitment's influence on user engagement (Zhao and Shi). Perceived support by the network manager derives not from commitment but from the value-sharing of performance. It is crucial for all partners in the network to feel that the network is beneficial for all the partners. Otherwise, the gap between the network manager and all other partners, such as consumers, users, communities, and local government, can result in only short-term successful performance. Therefore, our focus should move to the governance factors involved in this workable mechanism. Several studies have also addressed these issues.

## Sustainable governance in the turbulent economy

Free trade under the WTO regime has led to more benefits for developing countries to increase their exports to the one integrated global market. Owing to this borderless competition, China became the “global factory” with the lowest prices in the global market in the early 2000s. However, the sharp increase in expertise and its resulting economic development in BRICS and ASEAN countries contributed to the developed countries experiencing deep depression in their domestic economy. Impacts included BREXIT (the UK's exit from the EU) in Europe, and trade conflict between the present and emerging global leaders, the United States and China, respectively. The so-called winner's curse seems to have emerged in this turmoil between emerging countries and shrinking economies (Thaler, 1988). The global market has stumbled, and most countries have been experiencing deep recession. When the developed countries began to loosen their local financial markets by issuing “helicopter money” with zero interest rates, the severity of the recession increased. This money flooded into other developing countries to boost their property prices, resulting in hyperinflation worldwide. In this process, the countries must promote innovation challenges to overcome the turmoil of recession. In particular, environmental concerns under the Paris Regime have created new challenges regarding the carbon-zero economy to overcome the global climate crisis. All these new challenges require a paradigm shift from traditional efficiency maximization toward value creation for all partners in the collaborative network through green technology innovation or the smart ecology revolution. Is this feasible? This Research Topic considers the major concerns from the perspective of sustainable governance.

Zhuo et al. studied the impact of brand experience and its mechanism using data from 508 virtual sports brand communities and found that value co-creation (i.e., corporate- and customer-initiated value co-creation) has a positive effect on brand experience as well as on the purchase intention. They argued that the goal of the corporation is no longer to create customer value but to encourage customers to create the value they need from the services that are provided by the corporate entity. This is because the brand image supplied by the corporation is just superficial value for consumers, but invisible, yet perceivable service quality from the business performance of the corporation makes them feel loyalty as the sustainable value. This sustainable loyalty value is important in this turbulent era of a paradigm shift because it promotes the voluntary participation of all interested stakeholders to enhance the sustainable performance of the corporation. Thus, governance comes from the partnership in the collaborative network because each partner feels their contribution is valuable for all with sustainable performance. This partnership is workable not only in traditional manufacturing industries but also in IT industries. Computational thinking is now recognized as a foundational competency for students majoring in Science, Technology, Engineering, and Mathematics (STEM). Hsieh et al. examined the integration of tangible robots into project-based learning (PBL) courses of thinking skills training to improve the learning performance of students' computational thinking ability. Their results revealed that the PBL method integrated with the teaching material of the robotic visual programs approach was significantly more effective in improving students' learning achievements (Hsieh et al.). Partnerships, even with robotics, can enhance cognitive performance. Therefore, value-sharing partnerships play a crucial role in promoting governance even in this turbulent economy.

## Conclusion

K-Pop band Blackpink received honorary Member of the Order of the British Empire (MBEs) from Britain's King Charles on November 11, 2023. According to the UK government, this group received MBEs in recognition of the members' role as COP26 Advocates for the United Nations climate summit held in Glasgow in 2021 (Reuters, 2023). How did this happen? Because we live in a network society, global climate change is not a concern for one single economic agent, but for all people worldwide. This group promoted the cognitive concern on the climate crisis and the UK government wants to share these cognitive challenges as well by giving this award. The major role of the group is not to warn about the climate crisis, however, they are ready to share this innovative role as a network manager for the global ecology system. Their readiness to be network managers for the climate crisis can be a great impetus for people worldwide, including in the UK. This is the working mechanism of value-sharing based on network management, as highlighted in this Research Topic.

This Research Topic contains diverse research on sustainable governance in this transient, turbulent global economy. The most important key message on the issues is the partnership to share the risky responsibility as well as the resulting right to value-sharing. In the transition era toward the 4th Industrial Revolution supported by the mobile big bang as well as the environmentally friendly carbon-zero economy, the traditional approach to maximize the profits of corporations and/or maximize the economy with lower inputs and higher outputs is no longer workable because competitive solutions may hurt one of the future or potential partners. Innovation is the pressing need to overcome this traditional paradigm. Unfortunately, innovation does not originate from geniuses. Group intelligence is more valuable for innovative solutions for all. Therefore, the partnership is crucial to promote innovation in sustainable, smart technology because innovation comprises creative work undertaken on a systematic basis to increase the stock of knowledge, including knowledge of humankind, culture, and society, and the use of this stock of knowledge to devise new applications (Lee et al., 2020).

Then, who is going to take on the mediating role of this partnership? The role of network managers as mediators is the most important, as demonstrated in most of the papers in this Research Topic. In summary, the role of mediators should be based on three functions facilitator, collaborator, and one-stop service provider (Choi, 2018).

## Author contributions

YC: Conceptualization, Writing – original draft. S-CC: Investigation, Writing – review & editing. YM: Investigation, Writing – review & editing. AR: Investigation, Writing – review & editing.

## References

[B1] Choi Y. (2017a). Sustainable governance in Northeast Asia: challenges for the sustainable Frontier. Sustainability 9:191. 10.3390/su9020191

[B2] Choi Y. (2017b). Digital Business and Sustainable Development: Asian Perspective. New York, NY: Rotledge Co.

[B3] Choi Y. (2018). The Asian values of guanxì as an economic model for transition toward green growth. Sustainability 10:2150. 10.3390/su10072150

[B4] Lee H. Choi Y. Seo H. (2020). Comparative analysis of the R&D investment performance of Korean local governments. Tech. Forecast. Soc. Change 157:120073. 10.1016/j.techfore.2020.120073

[B5] Lin X. Suanpong K. Ruangkanjanases A. Lim Y.-T. Chen S.-C. (2022). Improving the sustainable usage intention of mobile payments: extended unified theory of acceptance and use of technology model combined with the information system success model and initial trust model. Front. Psychol. 12:634911. 10.3389/fpsyg.2021.634911 35082707 PMC8784512

[B6] Reuters (2023). K-Pop band BLACKPINK receive honorary MBEs from Britain's King Charles, internet news. Available online at: https://www.reuters.com/lifestyle/k-pop-band-blackpink-receive-honorary-mbes-britains-king-charles-2023-11-22/ (accessed November 23, 2023).

[B7] Thaler R. H. (1988). Anomalies: the winner's curse. J. Econ. Perspect. 2, 191–202. 10.1257/jep.2.1.19120305741

[B8] Wang Y. Zhao Z. J. (2018). Performance of public–private partnerships and the influence of contractual arrangements. Public Perform. Manag. Rev. 41, 177–200. 10.1080/15309576.2017.1400989

